# Purine nucleoside phosphorylase from *Schistosoma mansoni* in complex with ribose-1-phosphate

**DOI:** 10.1107/S0909049510027718

**Published:** 2010-11-12

**Authors:** Humberto D’Muniz Pereira, Glaucius Oliva, Richard Charles Garratt

**Affiliations:** a Centro de Biotecnologia Molecular Estrutural, Instituto de Física de São Carlos, Universidade de São Paulo, Avenida Trabalhador São-Carlense 400, CEP 13566-590, Brazil

**Keywords:** schistosomiasis, purine nucleoside phosphorylase, ribose-1-phosphate

## Abstract

Schistosomes are blood flukes which cause schistosomiasis, a disease affecting approximately 200 million people worldwide. Along with several other important human parasites including trypanosomes and *Plasmodium*, schistosomes lack the *de novo* pathway for purine synthesis and depend exclusively on the salvage pathway for their purine requirements, making the latter an attractive target for drug development. Part of the pathway involves the conversion of inosine (or guanosine) into hypoxanthine (or guanine) together with ribose-1-phosphate (R1P) or *vice versa*. This inter-conversion is undertaken by the enzyme purine nucleoside phosphorylase (PNP) which has been used as the basis for the development of novel anti-malarials, conceptually validating this approach. It has been suggested that, during the reverse reaction, R1P binding to the enzyme would occur only as a consequence of conformational changes induced by hypoxanthine, thus making a binary PNP–R1P complex unlikely. Contradictory to this statement, a crystal structure of just such a binary complex involving the *Schistosoma mansoni* enzyme has been successfully obtained. The ligand shows an intricate hydrogen-bonding network in the phosphate and ribose binding sites and adds a further chapter to our knowledge which could be of value in the future development of selective inhibitors.

## Abstract 2

A binary complex between a low-molecular-weight purine nucleoside phosphorylase and ribose-1-phosphate is described for the first time and comparisons with known ternary complexes are drawn.

## 1. Introduction

Schistosomiasis is the name of a severely debilitating disease caused by parasites of the genus *Schistosoma*, among which *Schistosoma mansoni* is the most prevalent species in the world and is estimated to infect approximately 200 million people (World Health Organization, 2005 ▶). The parasite shows a complete dependence on preformed purines for its metabolic needs owing to an absence of the enzymes which comprise the ‘*de novo*’ pathway for purine biosynthesis. Schistosome’s dependence on the purine salvage pathway for providing essential purines may therefore represent a metabolic ‘Achilles heel’ (Dovey *et al.*, 1984 ▶; Senft & Crabtree, 1983 ▶; Senft *et al.*, 1972 ▶).

One key component of this pathway is the enzyme purine nucleoside phosphorylase (PNP), which catalyzes the reversible phosphorolysis of the *N*-ribosidic bond of 6-oxopurine deoxynucleosides and nucleosides yielding their correspondent base and ribose-1-phosphate. Crystal structures of several PNPs have been described which can be assigned to two main categories: the low-molecular-mass homotrimeric PNPs which bind to 6-oxopurines, and high-molecular-mass homohexameric PNPs that accept either 6-oxo or 6-aminopurine (such as adenosine) as substrates (Bzowska *et al.*, 2000 ▶). PNP has been considered as an attractive target for drug development against several different parasitic infections as well as against T-cell proliferative cancers (Madrid *et al.*, 2008 ▶; Rinaldo-Matthis *et al.*, 2007 ▶; Schramm, 2004 ▶). Transition-state analogues with *K*_*i*_ in the n*M* to p*M* range have been developed for the proposed treatment of T-cell lymphoma (Clinch *et al.*, 2009 ▶; Rinaldo-Matthis *et al.*, 2007 ▶; Schramm, 2004 ▶; Chen & Advani, 2008 ▶) and several ground-state purine analogues have been assayed for their Schistosomicidal activity (el Kouni, 1991 ▶; el Kouni *et al.*, 1987 ▶; Dovey *et al.*, 1985 ▶).

An important step to improve the potency and selectivity of current inhibitors of PNP which might be useful against schistosomiasis is a more complete structural characterization of the enzyme’s catalytic mechanism. For instance, although over 100 PNP structures from 13 different organisms have been described to date, none of them describe the crystallographic structure of PNP as a binary complex with ribose-1-phosphate (R1P), one of the products of the phosphorolysis reaction. Currently, only an approximate description of this stage in catalysis is available from a ternary complex between bovine PNP, hypoxanthine and R1P [Protein Data Bank (PDB) code 1a9t] (Mao *et al.*, 1998 ▶). Following unsuccessful attempts to obtain a complex of bovine PNP with R1P, these authors hypothesized that R1P binding would occur as a consequence of conformational changes induced by hypoxanthine, thus making a PNP–R1P complex unlikely.

A second mechanistically important point that requires further elucidation is the importance of the conformational change described as the ‘gate movement’ which occurs upon substrate binding. This movement requires residues 257–265 in both human and bovine PNPs to shift from a coil to an α-helical conformation (Canduri *et al.*, 2004 ▶; Mao *et al.*, 1998 ▶). On the other hand, previous crystallographic studies of SmPNP have shown that the equivalent region (residues 259–267) in subunits *A* and *C* is already helical even in the absence of bound ligand (Pereira *et al.*, 2005 ▶). In subunit *B* this region is frequently disordered and only assumes an α-helical conformation in the presence of bound nucleoside while presenting an alternative extended structure in the complex with adenine (3e9r) (Pereira *et al.*, 2010 ▶). However, such analyses are complicated by the potential effects of crystal packing forces.

Aiming at shedding some further light on these questions we report for the first time the crystal structure of a binary complex between PNP and R1P alone. We make comparisons with other known complexes which serve to illustrate the progressive structural rearrangements of the low-molecular-weight PNPs required for binding and catalysis.

## 2. Materials and methods

### 2.1. Crystallization, soaking and X-ray data collection

SmPNP was expressed, purified and crystallized as previously described (Pereira *et al.*, 2003 ▶ 2005 ▶). The soaking solution consisted of 5 m*M* R1P, 20% PEG 1500, 15 m*M* sodium acetate buffer pH 4.9 or 5.0 (at the pH of crystal growth) and 20% glycerol. The crystals were maintained in this solution for up to 48 h.

X-ray diffraction data were collected at 100 K on beamline MX1 of the LNLS (Campinas, Brazil) using an X-ray wavelength of 1.43 Å. The crystals of the complex SmPNP–R1P diffract up to 2.0 Å resolution. The data were indexed and integrated using the program *MOSFLM* (Leslie, 1999 ▶) and scaled using the program *SCALA* from the CCP4 suite (Collaborative Computational Project, Number 4, 1994 ▶).

### 2.2. Structure solution and refinement

The SmPNP–R1P complex was solved by molecular replacement employing *MOLREP* (Vagin & Teplyakov, 2000 ▶) and using native SmPNP (1td1) as a search model (Pereira *et al.*, 2005 ▶). The refinement was carried out using *Phenix* (Adams *et al.*, 2002 ▶) together with *Coot* (Emsley & Cowtan, 2004 ▶) for model building into σ_a_-weighted 2*F*_o_ − *F*_c_ and *F*_o_ − *F*_c_ electron density maps. The compounds were automatically placed using the ‘Find Ligand’ routine of *Coot*; the water molecules were identified and positioned using both *Coot* and *Phenix*.

Both *R* and *R*_free_ were monitored in order to evaluate the validity of the refinement protocol and the stereochemical quality of the model was calculated using *Procheck* (Laskowski *et al.*, 1993 ▶). The coordinates and structure factors have been deposited in the PDB under the code 3fb1.

## 3. Results

### 3.1. Overall structure description

**Figure 1:**
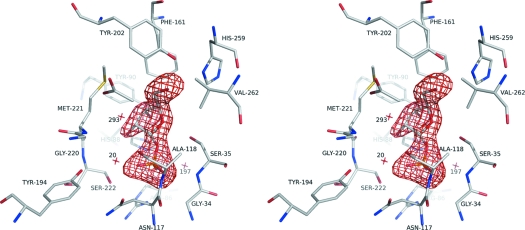
Stereo image of an omit map contoured at 3σ for ribose-1-phosphate in the active site of SmPNP (*A* subunit).

The asymmetric unit of the structure of SmPNP in complex with R1P (SmPNP–R1P) contains the full PNP trimer (6347 protein atoms) along with 368 water molecules, three molecules of R1P and three molecules of acetate. The final *R* and *R*_free_ values as well as other statistical parameters from the data collection and model refinement are listed in Table 1 ▶. All ligands, including three R1P molecules, were readily positioned in the *F*_o_ − *F*_c_ electron density map (Fig. 1 ▶) whilst residues for which there was little or no interpretable density were removed from the final structure (subunit *A*: residues 1–3 and 63–65; subunit *B*: 1–3 and 254–266; subunit *C*: 1–2).

### 3.2. Active site description

We describe here the first binary complex between PNP and the reaction product R1P. Previously, Mao and collaborators have described two ternary complexes of the bovine enzyme involving the R1P product, one in the presence of 9-deazahypoxanthine and the other in the presence of hypoxanthine (Mao *et al.*, 1998 ▶). These authors demonstrated that the bovine PNP crystals are catalytically active under the crystallization conditions used and that the enzyme can bind R1P in the presence of hypoxanthine. However, they were unable to obtain a complex of bovine PNP with R1P alone, even after exhaustive soaking experiments including up to 4 m*M* concentrations of the ligand. The authors concluded that R1P does not bind alone to the PNP active site but rather that this is a result of conformational changes induced by hypoxanthine binding which leads to an increased affinity for R1P.

Contrary to this hypothesis, we have been able to obtain the elusive binary complex of SmPNP with R1P (Fig. 1 ▶). It is interesting to speculate as to why we have been successful where previous attempts failed. One possibility is the time used during the soaking experiment, which was much longer than that reported previously (48 h compared with a maximum of 2 h used for bovine PNP). In the case of SmPNP, experiments in which shorter soaking times were used proved to be unsuccessful. Secondly it is worth pointing out that the PNP binding site is known to be extremely flexible and often presents differences even between subunits of a given crystal form. This would seem to suggest great sensitivity to crystal environment and packing forces which are known to influence the success of soaking experiments.

The ligand presents an intricate hydrogen-bonding network involving either ten direct and four water-mediated hydrogen bonds (subunit *A*) or nine direct and five water-mediated hydrogen bonds (sub­units *B* and *C*). These interactions are limited to the well defined phosphate and ribose binding sites which are lined by residues S35, R86, H88, Y90, A118, Y202, M221, S222 and H259. Overall, the structures of the three monomers are highly conserved, not deviating more than ∼0.2 Å RMSD from one another after Cα superposition.

The base binding site is occupied by one acetate molecule which interacts with E203 as described in previous SmPNP structures (Pereira *et al.*, 2005 ▶). Overall the phosphate and ribose binding sites of all three subunits are very similar, but two exceptions are worthy of mention. Firstly the loop from residue 250 to 266 in the *B* subunit cannot be seen in the 2*F*_o_ − *F*_c_ electron density map, as is frequently the case in other SmPNP structures. This suggests that the binding of R1P is not sufficient in itself to cause the loop rearrangement observed in complexes with the nucleosides inosine and adenosine. Even in subunits *A* and *C*, where the loop is well ordered, significant differences compared with the SmPNP–inosine structure can be seen. This is most notable for the interaction between O5′ of the ribose and H259 N_δ1_, which is weaker in the SmPNP–R1P complex (3.4 Å and 3.07 Å in subunits *A* and *C*, respectively, compared with 2.73 Å and 2.62 Å in the SmPNP–inosine complex).

**Figure 2:**
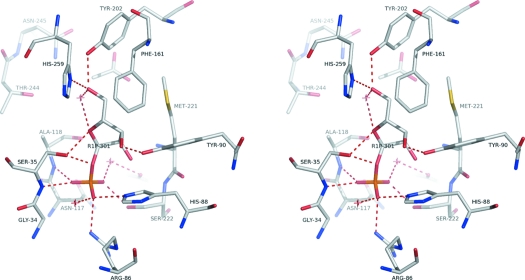
Stereo image showing the interactions made by ribose-1-phosphate in subunit *A* of SmPNP. The conformation of the phosphate binding loop, centred on residue S35, allows the formation of hydrogen bonds between Oγ of S35 and O1′ and O4′ of R1P as well as the main chain NH with O3P.

The second difference involves the phosphate binding loop (residues 33 to 37). In previously determined structures of SmPNP with inosine (3faz), adenosine (3f8w) and hypoxanthine (3fqn), in which the phosphate binding site is occupied by sulfate, this loop presents two different conformations in the three subunits. The SmPNP–R1P complex is the first structure in which we observe a unique conformation for this loop suggesting that the anionic moiety may need to be covalently bound to the ribose in order to fully favour all interactions made by this loop. These include those made simultaneously by Ser35 which include hydrogen bonds between Oγ and R1P O1′and O4′ and the main chain nitrogen with O3P (Fig. 2 ▶). This is supported by the observation of similar interactions made in the ternary complex of bovine PNP with R1P and hypoxanthine (1a9t) (Mao *et al.*, 1998 ▶). Our attempts to obtain a similar ternary complex in the case of SmPNP by soaking were unsuccessful, SmPNP–hypoxanthine complexes being obtained instead.

**Figure 3:**
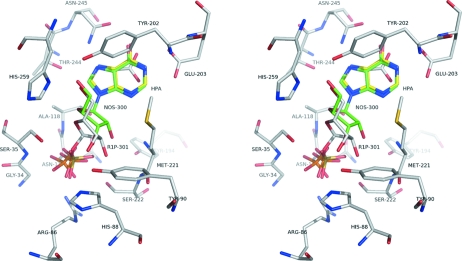
Superposition of the complexes between SmPNP and inosine/SO4 (green), hypoxanthine/SO4 (yellow) and ribose-1-phosphate (white). Protein residues are shown only for the SmPNP–R1P structure.

Fig. 3 ▶ shows simultaneously complexes between SmPNP and hypoxanthine/SO_4_, inosine/SO_4_ and R1P. The most notable difference seems to be the orientation of the ribose in the latter two complexes. This would seem to emphasize the comment made above that the connectivity between the phosphate and ribose is essential for correctly closing the phosphate binding loop. Together with the many different complexes described to date from several different species, the structure we present here therefore serves to complete the overall picture of flexibility within the PNP active site.

## Supplementary Material

PDB reference: 3fb1

## Figures and Tables

**Table 1:** Full data collection and refinement statistics for SmPNP–R1P The numerals in parentheses are from the highest-resolution shell.

Data collection	
Space group	*P*2_1_2_1_2_1_
Cell dimensions *a*, *b*, *c* (Å)	48.92, 117.55, 29.23
Detector	MarCCD 165
X-ray source	LNLS D03B-MX1
Wavelength (Å)	1.432
Resolution range (Å)	129.67–2.0 (2.11–2.0)
Redundancy	4.5 (4.7)
*R*_meas_ (%)†	7.5 (55.3)
Completeness (%)	95.1 (86.7)
Total reflections	341663 (15830)
Unique reflections	48547 (6312)
*I*/σ(*I*)	14.5 (2.0)

Refinement parameters	
Reflections used for refinement	45735
*R* (%)‡	17.7
*R*_free_ (%)‡	23.4
Overall averaged *B*-factor (Å^2^)	35.70
Ligand averaged *B*-factor (Å^2^)	36.98
No. of protein atoms	6347
No. of water molecules	368
No. of ligand atoms	54
Ramachandran plot	
Most favoured region (%)	90.8
Residues in disallowed regions (%)	0.4
R.m.s. bond lengths (Å)	0.014
R.m.s. bond angles (°)	1.223

† *R*_merge_ = Σ_*hkl*_Σ_*i*_|*I*_*i*_(*hkl*) − 〈*I*(*hkl*)〉|/Σ_*hkl*_Σ_*i*_ *I*_*i*_(*hkl*), where *I*_*i*_(*hkl*) is the observed intensity of the measured reflection and 〈*I*(*hkl*)〉 is the averaged intensity over equivalent reflections from different measurements.

‡ *R* is the conventional crystallographic *R*-factor, Σ||*F*_obs_| − |*F*_calc_||/Σ|*F*_obs_|, where *F*_obs_ and *F*_calc_ are the observed and calculated structure factors, respectively. 5% of the reflections that were excluded from the refinement were used in the *R*_free_ calculation.
